# New Plant Breeding Techniques in Citrus for the Improvement of Important Agronomic Traits. A Review

**DOI:** 10.3389/fpls.2020.01234

**Published:** 2020-08-14

**Authors:** Fabrizio Salonia, Angelo Ciacciulli, Lara Poles, Helena Domenica Pappalardo, Stefano La Malfa, Concetta Licciardello

**Affiliations:** ^1^ CREA - Research Centre for Olive, Fruit and Citrus Crops, Acireale, Italy; ^2^ Department of Agriculture, Food and Environment (Di3A), University of Catania, Catania, Italy

**Keywords:** *Citrus*, editing, cisgenesis, fruit quality, transformation, regeneration, marker-free vectors, early flowering

## Abstract

New plant breeding techniques (NPBTs) aim to overcome traditional breeding limits for fruit tree species, in order to obtain new varieties with improved organoleptic traits and resistance to biotic and abiotic stress, and to maintain fruit quality achieved over centuries by (clonal) selection. Knowledge on the gene(s) controlling a specific trait is essential for the use of NPBTs, such as genome editing and cisgenesis. In the framework of the international scientific community working on fruit tree species, including citrus, NPBTs have mainly been applied to address pathogen threats. Citrus could take advantage of NPBTs because of its complex species biology (seedlessness, apomixis, high heterozygosity, and long juvenility phase) and aptitude for *in vitro* manipulation. To our knowledge, genome editing in citrus *via* transgenesis has successful for induced resistance to Citrus bacterial canker in sweet orange and grapefruit using the resistance gene CsLOB1. In the future, NPBTs will also be used to improve fruit traits, making them healthier. The regeneration of plants following the application of NPBTs is a bottleneck, making it necessary to optimize the efficiency of current protocols. The strengths and weaknesses of using explants from young *in vitro* plantlets, and from mature plants, will be discussed. Other major issues addressed in this review are related to the requirement for marker-free systems and shortening the long juvenility phase. This review aims to summarize methods and approaches available in the literature that are suitable to citrus, focusing on the principles observed before the use of NPBTs.

## Introduction

Citrus belongs to *Rutaceae* family and is among the most important fruit crops in the world. Citrus fruits represent a source of macro- and micronutrients (Ting, 1980) and of dietary fiber (Marín et al., 2007). They are also rich in antioxidants compounds (Liu et al., 2012), reveal anticancer, and anti-inflammatory properties (Reviewed in Ma et al., 2020), and are effective at reducing the risk of cardiovascular disease, osteoporosis, and type-2 diabetes (Yuan et al., 2003; Tripoli et al., 2007; Benavente-García and Castillo, 2008; Sugiura et al., 2012; Cirmi et al., 2016; Sugiura et al., 2016).

Citrus, like other woody plants, has a long juvenile phase, extensive hybridization and outcrossing, and reduced population structure. Most cultivated citrus species were domesticated from their wild ancestors or underwent crossbreeding. Recently, an increasing number of studies have focused on the domestication of *Citrus*, helping us to elucidate the origin of cultivated species. This provides a comprehensive resource for how wild resources can contribute to improve the existing varieties (Wu et al., 2014; Wang et al., 2017; Wu et al., 2018; Ahmed et al., 2019).

Traditional breeding is one of the main strategies used to improve agronomic traits. In many *Citrus* species, several varieties have developed through conventional methods, such as mutagenesis, inter- and intra-specific crosses, and clonal selection (Caruso et al., 2020). The aim is to produce high-quality citrus fruits (in terms of size, sugar and acidity balance, juice yield, and seedlessness), that are healthy and rich in antioxidant compounds, tolerant or resistant to different abiotic and biotic threats, and with high productivity. Conventional citrus breeding is a long-term and expensive process; long time and resources to obtain progenies and to evaluate their traits are needed. In addition, sexual breeding is not always feasible because some cultivars to be used in crosses are incompatible, sterile, or polyembrionic (Talon and Gmitter, 2008). Moreover, in many cases, after breeding, backcrosses are required to recover elite features of the improved cultivar, lengthening even more breeding programs. This process can be also longer in the case of rootstock breeding (25 years and more). Despite their relatively low efficiency, traditional breeding methods so far enabled the release of most of the new varieties and rootstocks in citriculture.

Since the 1990s, new biotechnology techniques including the use of molecular markers, genome mapping, sequencing, and *in vitro* culture have been applied to breeding, providing efficient alternatives to traditional methods for the improvement of novel varieties. In addition, transgenesis had enabled the release of many commercial varieties and new rootstocks in citriculture (Gentile et al., 2007; La Malfa et al., 2011), in particular, with improved resistance to biotic and abiotic stresses. This has been possible through the development of transformation protocols involving *Agrobacterium tumefaciens* infection or polyethylene glycol (PEG) mediated DNA uptake process, starting from many source of explants, such as epicotyls and internodes or embryogenic cell suspensions and protoplasts.

**Figure 1:**
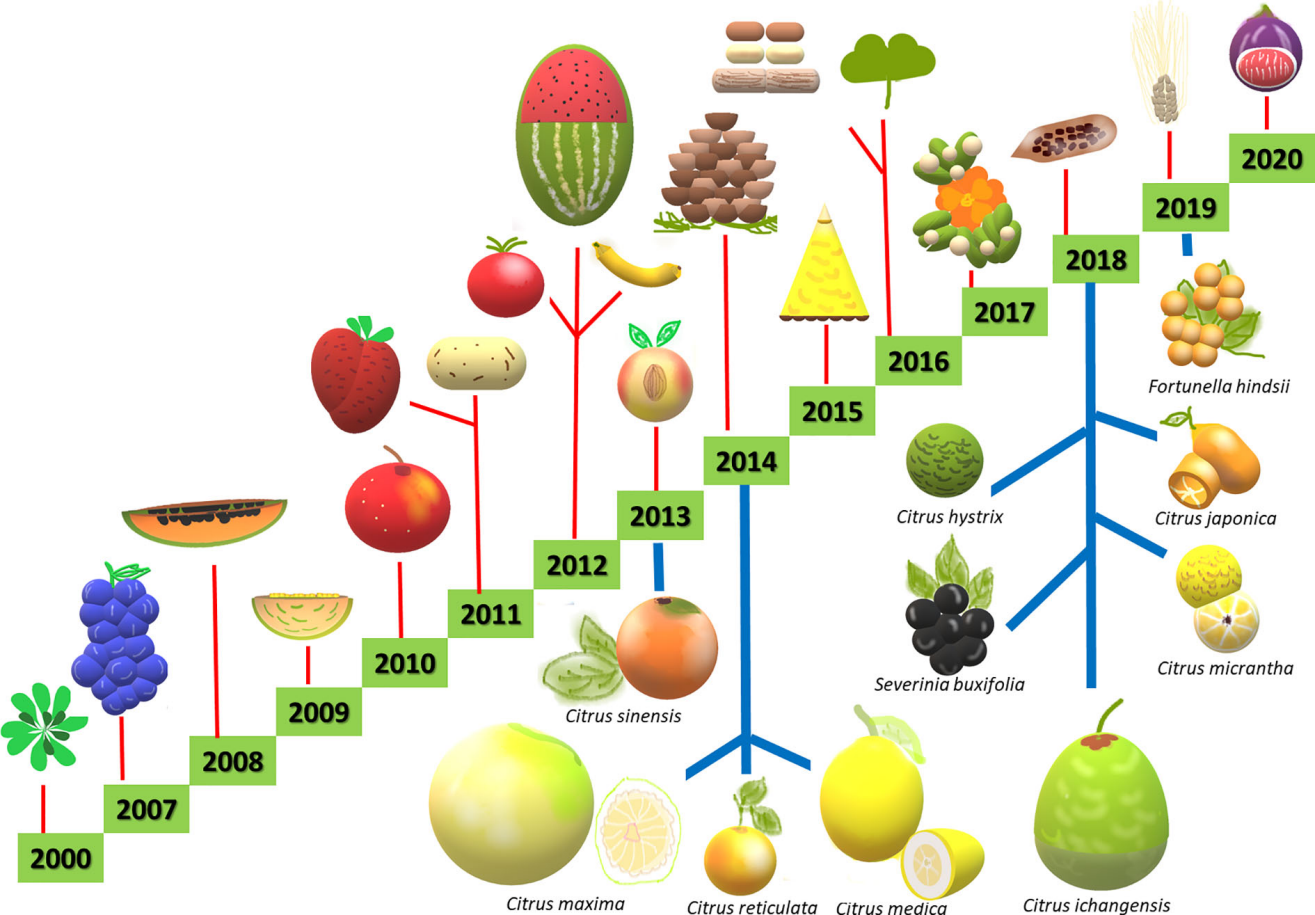
Some of the main plant genomes sequenced from years 2000 to 2020, including *Citrus* and related genera (bottom, indicated with blue lines) and other crops (up, indicated with red lines).

**Figure 2:**
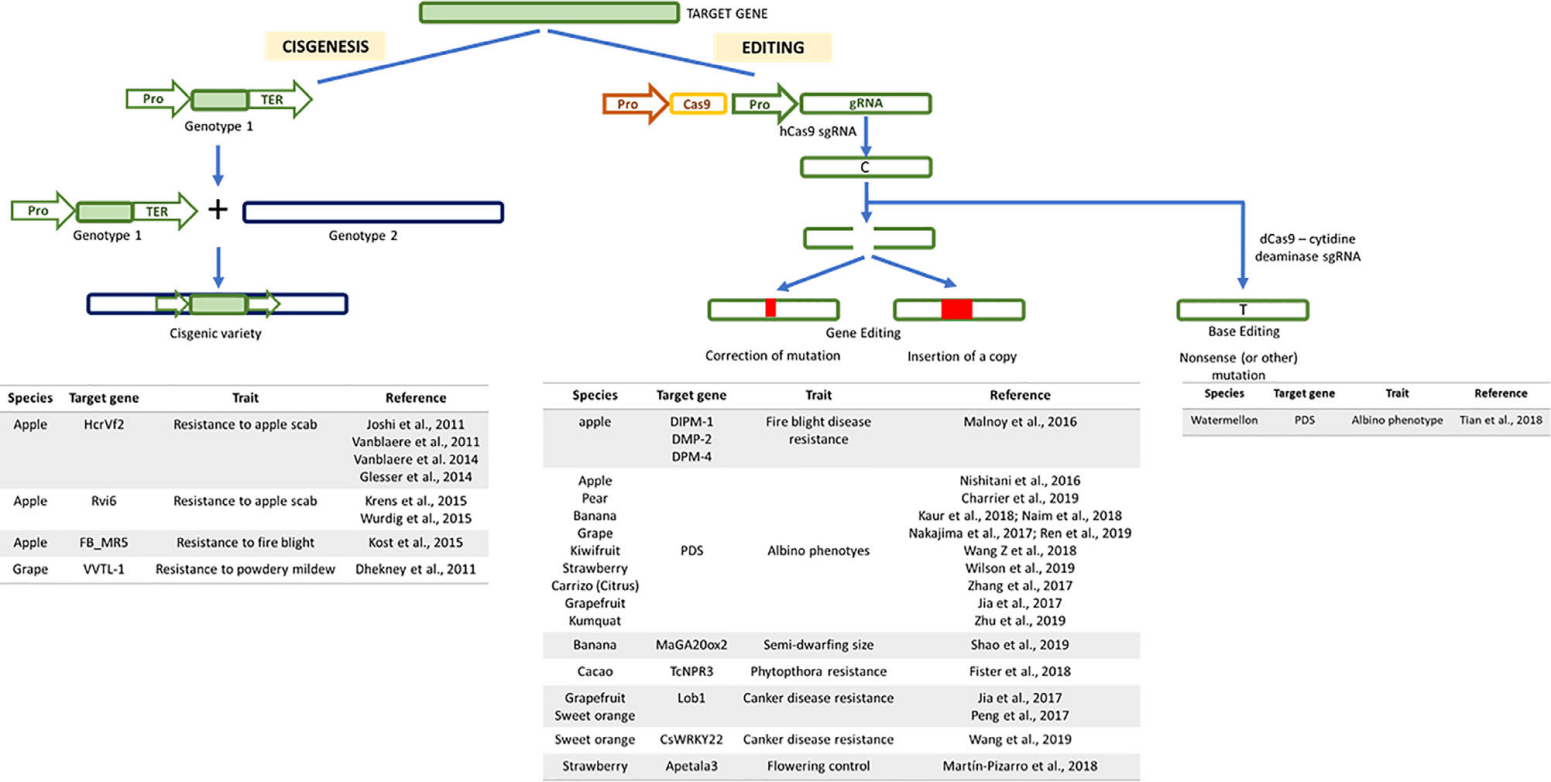
Genome modifications obtained by the application of new plant breeding techniques approaches illustrated through schematic workflow. In “cisgenesis” the new trait is derived from a sexually compatible species and it is transferred as it is, with its promoter and terminator, to the recipient. The “gene editing” requires the use of a Cas9 to modify the guide RNA (gRNA), specifically designed on the target gene. “Base editing” consists in the action of a chimeric protein composed of a dead or nickase Cas9 with a conserved specific DNA binding capacity and a base modifying enzyme such as adenosine and cytidine deaminase. Below each approach, a list of the main target genes controlling specific traits of several fruit tree crops are reported with corresponding references.

More recently, a number of new techniques has been developed and classified as new plant breeding techniques (NPBTs). These include (I) zinc finger nuclease technology, (II) oligonucleotide directed mutagenesis, (III) cisgenesis, (IV) intragenesis, (V) RNA-dependent DNA methylation, (VI) grafting on genetically modified rootstock, (VII) reverse breeding, (VIII) agro-infiltration, and (IX) synthetic genomics (Lusser et al., 2011). However, the efficiency of NPBTs requires knowledge on the genetic control of horticulturally important traits, which remains limited in citrus compared to other major crops. In the last 20 years, the development of different technologies and sequencing platforms has resulted in the publication of genomes from several horticultural species (**Figure 1**). Regarding woody plants, to date, one of the main applications of NPBTs is the improvement of agronomic traits related to biotic and abiotic stress resistance (**Figure 2**). So far, NPBTs are ruled as genetically modified organisms (GMO) according to the GMO 2001/18 legislation, because they are included among the recombinant DNA technologies. In Europe several debates are ongoing in order to establish if the NPBTs methodologies themselves, or their products, could undergo to different protocols for their acceptance, autonomous from GMO regulation.

The objective of the present review was to describe the current state of the art and recent advances on the use of NPBTs, in particular regarding genome editing and cisgenesis, on citrus, and other fruit tree species. We highlighted technical, applicative, and legislative aspects of these technologies and the potential advantages and disadvantages of applying NPBTs to improve qualitative traits of fruit, making them healthier due to a higher content of antioxidants.

## Current Status of New Plant Breeding Techniques Worldwide

NPBTs provide alternative methods for advancing biotic and abiotic resistance, nutritional quality, and crop performance (Cao et al., 2016; Lassoued et al., 2018); among the others, genome editing and cisgenesis represent two of the most promising strategies to develop genetically improved tree crops. Genome editing involves the production of specific, stable, and inheritable mutations in a precise position of the genome, through DNA repair systems in the cell, with a low probability of inducing undesired errors (off-target effects) without leaving exogenous DNA. Cisgenesis involves the transfer of genes resulting from cross-compatible species. NPBTs represent innovative alternative methods to conventional breeding, with a shortened process (although this is true exclusively for tree fruit and woody plants), and for their precise mechanisms of action. NPBTs produce targeted and minimal modifications to selected genotypes, such as elite cultivars, which are highly valued by consumers for their quality and productivity but that can be even further improved. Unlike traditional breeding, and similarly to transgenesis approaches, NPBTs do not alter the genetic background and this is particularly important for elite cultivars. Advances in *in vitro* culture, in genome sequencing, and in functional studies have helped to improve the application of molecular breeding techniques in many crops. Many of the methodologies and protocols that make the use of NPBTs in plants feasible, including the availability of engineered plasmids, efficient *Agrobacterium*-mediated transformation and regeneration protocols from mature plants have been developed for the transgenic approach.

Recently, European and non-European countries, independently or coordinately, have supported the use of NPBTs to improve traits of several crops. GENIUS (2012–2019, https://www6.inra.genius-project_eng/) and BIOTECH (2018–2021, https://www.politicheagricole.it/flex/cm/pages/ServeBLOB.php/L/IT/IDPagina/9613) are projects in France and Italy, respectively, which showcase current NPBTs applied to traditional local crop plants. GENIUS addresses the use of genome editing for traits involved in the reduced use of pesticides and in the mitigation of climate change for more sustainable agriculture. The traits actually under evaluation used as proof of concept consist of resistance to biotic and abiotic stresses, flowering time and plant architecture control, plant reproductive mechanisms in several herbaceous, horticultural, ornamental, and fruit species, such as rice, wheat, maize, tomato, potato, oilseed rape, rose, poplar, and apple). BIOTECH is focused on the application of genome editing and cisgenesis to improve fruit qualitative traits, resistance to biotic and abiotic stress, and the architecture of main Mediterranean crops, such as rice, wheat, barley, tomato, eggplant, basil, grape, citrus, peach, apple, and poplar. GENIUS is founded by the French National Research Agency, while BIOTECH is funded by the Italian Agricultural Ministry. Moreover, Russia has also announced a proximal federal program aiming to create 10 new varieties of gene-edited among crops and animals within 2020, and another 20 crops by 2027 (Dobrovidova, 2019). Conversely, a Dutch project (2006–2016, https://www.wur.nl/en/Research-Results/Research-Institutes/plant-research/DuRPh.htm), DuRPh (durable resistance against *Phytophthora infestans*), aims to create cisgenic potatoes carrying multiple late blight-resistance genes from crossable wild species. In the framework of a European panel, PlantED (2019–2023, https://plantgenomeediting.eu/ CA18111) is the cost action aiming to assess the full innovation potential and impact of plant genome editing, setting the future direction of research priorities, promoting the link between research and innovation in a socially responsible manner, and examining the synergistic interactions between closely related fields. Moreover, also the International Community is working for developing short, medium, and long-term strategies in order to prevent and, eventually, to face the risk related to Huanglongbing (HLB), representing the most devastating citrus disease in the world. The identification of resistance and susceptible genes and the application of NPBTs to produce, in the near proximal future, resistance citrus plants represent only some of the objectives of the Horizon 2020 Project “preHLB”—preventing HLB epidemics for ensuring citrus survival in Europe (2019–2023). Finally, the United States Department of Agriculture (USDA) has financed projects aiming to engineer nanomaterials, or to identify methods to deliver CRISPR-Cas9 vectors and/or ribonucleoproteins (RNPs) to the plant nucleus, aiming to speed up the development of new crop varieties (https://nifa.usda.gov/program/plant-breeding-genetics-genomics-programs).

The regulation of NPBTs has been a topic of worldwide discussion. A critical point of discussion involves two aspects, considering either the used method or the characteristics of the end product. The resolution of critical issues on both aspects enables to process of NPBT or product assimilated to be considered, and, as consequence, ruled as a GMO or not. Eckerstorfer et al. (2019) analyzed regulatory frameworks for GMOs in different countries, and evaluated whether process- or product-addressed NPBT is more helpful for the regulation of NPBT applications. This led to the conclusion that neither system can be regarded as superior. In addition, NPBTs are differentially regulated throughout the world (Eckerstorfer et al., 2019; Friedrichs et al., 2019). In the USA, NPBT crops are exempt from the strict rules and regulations of the USDA (Waltz, 2016), considering genome editing as a method of conventional breeding, only faster. However, the USDA declared that products resulting from genome editing approaches should be considered on a case by case basis (Lusser and Davies, 2013; Voytas and Gao, 2014; Jones, 2015). Conversely, in Europe, the Court of Justice of the European Union (CJEU) (case C-528/16, ruling issued 25th July 2018) declared that NPBTs should be considered GMOs. In this way, NPBTs are subjected to the obligations of Directive 2001/18/EC, whereby GMOs are organisms whose genetic material has been altered and does not derive by natural reproduction and/or recombination.

### Examples of the Use of Cisgenesis in Fruit Tree Species

The term cisgenesis was first introduced by Schouten et al., in 2006, who defined it as “the genetic modification of plants using genes that originate only from the species itself or from a species that can be crossed conventionally with this species.” According to the above definition, cisgenesis involves the transfer of a gene (introns included) along with its controlling sequences (promoter and terminator, in the sense direction) from one genotype to another of the same or of a sexually compatible species (Schouten et al., 2006; Lusser and Davies, 2013). Cisgenesis can overcome the major bottleneck of traditional breeding, termed the ‘linkage drag’ (unwanted gene transfer along with the gene of interest), allowing the transfer of the gene of interest without other genetic regions controlling undesirable traits (Jacobsen and Schouten, 2007). Therefore, the gene pool considered by cisgenesis can also be theoretically transferred through classical breeding approaches (Holme et al., 2013). However, cisgenesis has several drawbacks, which limit its wider application. In particular, the casual insertion of the cisgene in the host genome could induce a negative effect (Vanblaere et al., 2014) and potentially interrupt or modify genic or intergenic relevant sequences. The many deposited genomes give information on genes and related annotations that can be used for the cisgenic approach; however, in many cases, the lack of efficient promoters and selectable markers remain the main bottleneck in the application of this technology (Limera et al., 2017). Moreover, the number of gene copies that will be integrated into the host genome may represent an additional drawback, even if, as for transgenesis and intragenesis, any clear correlation has been reported (Jones et al., 1987; Zanek et al., 2007; Zeng et al., 2009; Joshi et al., 2011). To date, few examples of cisgenic plants have been reported, and these are found almost exclusively in apple and grape, and aim to induce resistance to scab (Joshi et al., 2011; Vanblaere et al., 2011; Vanblaere et al., 2014; Gessler et al., 2014) and fire blight in apple (Krens et al., 2015; Würdig et al., 2015), as well as powdery mildew in grape (Dhekney et al., 2011) (**Figure 2**).

### Techniques and Methodologies for Genome Editing

Genome editing technologies represent effective tools for the accurate handling of targeted sequences for crop improvement (Arora and Narula, 2017; Limera et al., 2017). These technologies allow to edit, delete, and replace a specific sequence within a target site of a genome site without introducing extra DNA. These technologies induce double-strand breaks (DSB), then “repaired” through non-homologous end-joining (NHEJ) and homology-directed repair (HDR) processes (Gaj et al., 2013; Voytas, 2013; Fauser et al., 2014; Rinaldo and Ayliffe, 2015). Zinc finger (ZF) and transcription activator-like effector (TALE) represent first engineered nucleases adopted for targeted mutagenesis. These nucleases recognize specific target DNA sequences of one (TALE) or three (ZF) nucleotides, providing a nuclease that disrupts DNA adjacent to the recognition zones (Gaj et al., 2013). CRISPR/Cas9 (clustered regularly interspaced short palindromic repeat-associated protein 9) represents a revolutionary molecular tool that was originally discovered for defense against viral infection (Mojica et al., 2000). This technology works through guide RNAs (gRNAs), characterized by specific sequences designed on the basis of their targets in the genome. The Cas nuclease (commonly indicated as Cas9), when guided by gRNA, produces a DSB adjacent to the gRNA annealing location, permitting target-specific mutagenesis. CRISPR/Cpf1 (another CRISPR/Cas system) is more efficient (Ledford, 2015; Zetsche et al., 2015; Fonfara et al., 2016; Jia et al., 2019), overcoming several Cas9 limitations.

In plants, naturally occurring DNA double-strand breaks occur, and they are rejoined predominantly by NHEJ in absence of foreign donor sequences. This process is prone to errors, determining small mutations (such as frameshift caused by insertions and deletions) in the original sequence and inducing, as a consequence, the loss of function of the target gene and eventually a mutated phenotype. This is similar to the process of induced mutagenesis (by chemical or physical mutagens); however, in the genome editing approach, the induced mutations are not random (as in classical mutagenesis) but limited to specific genes of known function. Moreover, in conventional random mutagenesis, in addition to unwanted mutations, a large-scale screen of mutagenized populations is also needed to detect plants carrying mutations of interest. Conversely, CRISPR/Cas9 can also be associated with specific DNA fragments homologous to the target sequences.

One of the key advantages of CRISPR/Cas9 technology consists in its (relatively) ease of engineering, with modification of only 17–20 bp of the CRISPR RNA (crRNA), part of the guide RNA (gRNA), specificity, and complementary to the target DNA of the gene of interest. Several tools are available to assist with gRNA design, and for the *in silico* assembly of the construct to be used for plant transformation. Some of the most commonly used tools are Benchling (www.benchling.com), GoldenBraid 4.0 (www.gbcloning.upv.es), CRISPR-P 2.0 (www.crispr.hzau.edu.cn/cgi-bin/CRISPR2/CRISPR), CRISPRdirect (www.crispr.dbcls.jp), Chop-Chop (www.chopchop.cbu.uib.no), E-CRISPR (www.e-crisp.org), CRISPR-GE (www.crdd.osdd.net/servers/crisprge), CRISPR RGEN Tools (http://www.rgenome.net), and CRISPOR (http://www.crispor.tefor.net).

However, the use of CRISPR/Cas9 is limited by the occurrence of off-target mutations, which can generate unintended genetic changes in other regions of the genome. This phenomenon is likely due to the non-specific design of the gRNA and represents a critical limiting factor considering that Cas9 tolerates mismatches between gRNA and target DNA at different positions in a sequence-dependent manner (Hsu et al., 2013). Several tools, such as Cas-OFFinder (Bae et al., 2014) and VARSCOT (Wilson et al., 2019b), assist researchers regarding the location and number of potential off-targets that may hinder the genome editing approach. However, this approach can only be used for species with available genome information. Recently, Hajiahmadi et al. (2019) reported and discussed various methods addressed to reduce such off-targets without the use of external elicitant factors (such as temperature-independent methods), or expensive equipment.

To improve CRISPR/Cas technology and reduce limitations several different Cas, which differ in the position and sequence of the protospacer adjacent motif (PAM) recognition site, have been identified. Among these, Cas9 orthologs from *Streptococcus thermophilus* (St1Cas9) and *Staphylococcus aureus* (SaCas9) (Steinert et al., 2015), and Cas12a (formerly named Cpf1), which was identified in *Francisella* and *Pretovella* bacteria (Zetsche et al., 2015), are the most common.

To date, the main traits modified through gene editing are related to the albino phenotype, which is used as a proof-of-concept for established protocols, since it allows early phenotype screening. The phytoene desaturase (*PDS*) gene has been modified in many species, such as apple (Nishitani et al., 2016), pear (Charrier et al., 2019), banana (Kaur et al., 2018; Naim et al., 2018), grape (Nakajima et al., 2017; Ren et al., 2019), kiwifruit (Wang et al., 2018), strawberry (Wilson et al., 2019a), and citrus (Jia et al., 2017a; Zhang et al., 2017; Zhu et al., 2019). In addition to the albino trait, gene editing has been used to induce resistance to important pathogens for some fruit tree species (**Figure 2**). *Erwinia amylovora* is the causative agent of fire blight, an invasive disease that threatens apple and several commercial and ornamental Rosaceae. Editing the *DPM*-1, *DPM*-2-, and *DPM*-4 genes, which are disease-specific genes that provide susceptibility to *E. amylovora*, allowed the release of apple resistant genotypes to the disease (Malnoy et al., 2016). In the framework of fungal diseases, editing of cacao non-expressor of pathogenesis-related 3 (*NPR*3) gene, a negative regulator which represses the immune system of the plant, increased the resistance in planta to *Phytophthora tropicalis* (Fister et al., 2018). Furthermore, the Cas9 protein can be engineered (Fauser et al., 2014), and with this aim, two different enzymes with modified nuclease activity have been produced: nickase Cas9 (nCas9) and dead Cas9 (dCas9). nCas9 lacks nuclease activity in one between RuvC or HNH domains, retaining the ability to cleave only one strand, producing site-specific nicks (Fauser et al., 2014). dCas9 has lost its nuclease activity due to a point mutation in either the RuvC and HNH domain resulting in a loss of ability to break DNA. In this way, dCas9 exclusively recognizes target DNA without inducing double (or single) strand breaks (Qi et al., 2013). In 2016, a cytidine deaminase was fused with dCas9 and nCas9 to produce a cytosine base editor (CBE) that changes C:G to T:A (Hua et al., 2019). Similarly, Gaudelli et al., 2018 reported adenine base editors (ABEs) converting A.T to G.C. In this way, the mutated Cas-base editors produce single mutations in the target DNA specifically and precisely (base editing). Base editing has been adopted in plant species (**Figure 2**), such as watermelon (Li et al., 2017; Lu and Zhu, 2017; Ren et al., 2018; Tian et al., 2018), to produce transgene-free homozygous *als* (acetolactate synthase) mutant plants, resistant to the herbicide tribenuron (Tian et al., 2018). In addition to base editing, other applications of CRISPR/dCas9 in plants have been recently reviewed, including gene induction and suppression, epigenetic variations, and DNA-free modifications (Moradpour and Abdulah, 2020). More recently a highly versatile and accurate method has been developed, determining the presence of new genetic information in a specific DNA site. This approach is based on the use of nCas9 merged with a reverse transcriptase, engineered with a prime editing guide RNA (pegRNA). Both nCas9 and pegRNA specify the target site and encode the desired edit, inducing lower levels of off-target editing compared with Cas9 nuclease (Anzalone et al., 2019). To our knowledge, prime editing has been applied on annual crops, resulting in 21.8% prime-edited regenerated plants (Lin et al., 2020).

### Contribution of Horizontal Gene Transfer

Mutation mapping approaches have shown how similar mutations in orthologue genes among species that are not genetically related, exert similar effects on the phenotype. For example: i) the *PETALOSA* gene, when mutated in the microRNA172 target site, induces a dominant double flower phenotype in peach, rose, and carnation, and the phenotype has been already reproduced in tobacco by genome editing (Gattolin et al., 2020); ii) the *ALS* gene is the target gene of one of the most widely used herbicide classes, which inhibits the *ALS* enzyme. Many species developed independent resistance induced by a point mutation in the *ALS* sequence, with minimal effect on the catalytic function of the enzyme (Tranel and Wright, 2002). This knowledge allowed to replicate a point mutation in the *ALS* gene, by base editing, to produce herbicide-resistance crops (Shimatani et al., 2017); iii) finally, attention has been paid to the Terminal Flower gene family (*FT*/*FTL*), whose function is to guide the transition from vegetative to floral meristem and is preserved in many species (Pillitteri et al., 2004). Horizontal gene know-how transfer in fruit tree species has resulted in the production of marker-free vectors and in the reduction of juvenility based on *ALS* resistance and *FT*/*FTL* manipulation, respectively, as discussed in the next paragraphs.

### Marker-Free Vectors and the Development of New Reporter Genes

To be scientifically useful and relevant, modifications induced through NPBTs should be permanent in the plant. The main limitation in the use of stable transformants is the presence of selectable marker genes (SMGs), which can confer resistance to a toxin (in the case of antibiotics) or can metabolize specific products required by cells for growth. The use of marker genes for resistance to antibiotics (i.e., *nptII*) or herbicides is not accepted by the public, and, consequently, since December 31 2004, the European Food Safety Authority has forbidden the use of resistance markers antibiotics in Europe (Directive 2001/18/EC), in contrast to the USA and other countries. In annual crops “host” genes, deriving from a genome editing approach, can be discarded using traditional crosses; on the contrary this method is rather impossible in perennial species, due to the long time period needed, and the cross itself will not maintain the identity of the clonally propagated variety (Song et al., 2019a).

To overcome the limits associated with GMOs, several strategies have been tried to eliminate marker genes, consisting in the use of marker-free constructs and recombination systems to excise SMGs (Krens et al., 2015). In the first case, a gene editing can be performed using transient transformation without stable integration of the CRISPR/Cas9 components (Chen et al., 2018), although identification of targeted mutant cells is challenging due to a lack of SMGs for transformed plant cells (Song et al., 2019a). In these cases, it is essential to produce a large number of regenerants so to make it possible a large-scale selection; however, in most of fruit tree species the lack of efficient protocols represents a limiting factor (Rugini et al., 2020). In the second approach the marker gene, bordered by two recognition sequences for a recombinase, can be chemically (using dexamethasone) or physically (by heat-shock) activated, allowing the excision of SMGs. Several recombination loci bordering the unwanted sequences are available: cre-lox (Dale and Ow, 1991), R/Rs (Schaart et al., 2004), and FLP/FRT (Lyznik et al., 1993). Recombination/excision systems have been shown to effectively produce SMG-free apple (Kost et al., 2015; Krens et al., 2015), apricot (Petri et al., 2012), and citrus (Zou et al., 2013). Recently, Pompili et al. (2020) reported the use, in apple, of a CRISPR/Cas9-FLP/FRT gene editing system aimed at the production of regenerated plants with low content of residues of exogenous DNA.

Another attempt to produce marker-free plants was made in citrus (Ballester et al., 2008) and apricot (López-Noguera et al., 2009) consisting in the use of the multi-auto-transformation (MAT) vector associated with the isopentenyl transferase (*ipt*) gene for positive selection due to site-specific recombination. This method involves the removal of undesired sequences, resulting in the integrated of useful genes only. In citrus, 65% efficiency was demonstrated for ‘Pineapple’ sweet orange plant; however, the results were not appropriate for the ‘Carrizo’ citrange, due to the genotype specificity of this approach (Ballester et al., 2008).

Rather than removing integrated exogenous DNA, an approach to avoid the stable integration of foreign DNA was recently proposed. Thus, in several annual crops, transgene-free plants were produced directly by inducing point mutations through base editing in the gene of interest and in the ALS gene. The latter confers stable resistance to the herbicide, such that selection made by the selective agent, versus no-edited plants, does not impose any integration of foreign DNA. This approach is limited to traits reproducible by a point mutation (Danilo et al., 2019; Veillet et al., 2019; Zhang et al., 2019).

There has also been progress in the development of reporter genes belonging to the *MYB* transcription factor (TF) family, specifically involved in the activation of anthocyanin pigmentation in plants (Elomaa et al., 2003). In particular, Kandel et al. (2016) compared *VvMybA1* of grapevine with conventional reporter genes *GFP* and *GUS*, showing that the *MybA1* (used as reporter gene) is appropriate to evaluate the gene expression at cellular level. Similarly, in citrus Dutt et al. (2018b) used the embryo-specific *Dc3* gene promoter to drive the *VvMybA1*, inducing a anthocyanin-color based selection.

## State of The Art On Citrus

### Applications of New Plant Breeding Techniques in Citrus

Most cultivated citrus species originated from a complex admixture between four basic or primary species: citron (*Citrus medica*), pummelo (*Citrus maxima*), mandarin (*Citrus reticulata*), and *Citrus micrantha* (Xu et al., 2013; Velasco and Licciardello, 2014; Wu et al., 2014; Curk et al., 2016; Wu et al., 2018). Within the primary and derived species (including sweet orange and lemon), most of the worldwide cultivated citrus varieties are derived from somatic mutations of a single ancestor, which accumulated over time in the different growing areas (Wu et al., 2014; Caruso et al., 2016; Curk et al., 2016). Traditional breeding methods used for genetic improvement of citrus cultivars and create new varieties, or to improve desired traits require a long time and is hampered by many difficulties and restrictions related with plant size and long juvenile phase of citrus, as well as female and/or male sterility, polyembryony, heterozygosity, and parthenocarpy.

The partial availability and knowledge of the genes that control the traits of interest, has slowed recently, and the possibility to explore and make use of NPBTs in citrus is feasible. The genes and markers reported herein refer to well-known and characterized traits, and information on these genes and markers were developed after the availability of the *Citrus* genomes (Xu et al., 2013; Wu et al., 2014; Curk et al., 2016; Wang et al., 2017; Wu et al., 2018). To date, Ruby (a MYB-like TF) and its promoter 3′LTR, represents one of the main genes responsible for the control of purple pigmentation due to the presence of anthocyanins in citrus fruits (Butelli et al., 2012); *CsLOB1* (lateral organ boundaries gene family) and *CsWRKY22* are genes associated with susceptibility to citrus bacterial canker (CBC) in sweet orange (Hu et al., 2014; Wang et al., 2019); *CitRWP* controls polyembryony in sweet orange, grapefruit, lemon, and mandarin (Wang et al., 2017); *Noemi* represents Myc-like, which along with *Ruby*, controls anthocyanins pigmentation in *Citrus* and is also responsible for the acidless trait in citrus fruits (Butelli et al., 2019).

As for other fruit tree species, the application of NPBTs in *Citrus* has mainly focused on the generation of plants resistant to biotic stresses (varieties and rootstocks). Thus, the successful application of genome editing in citrus was first based on the use of *CsLOB1*, the gene responsible for susceptibility to CBC (Hu et al., 2014; Duan et al., 2018). CBC is a severe quarantine disease caused by the bacterium *Xanthomonas citri* pathovar *citri* (*Xcc*) and *aurantifolii* (*Xca*), which are found globally, except for the Mediterranean basin (Gottwald et al., 2001). Genetic engineering is the best approach to induce resistance to CBC (Zhang et al., 2010). Considering the difficulty to introgress resistance through traditional breeding, a recent method involved the use of NPBTs to intervene in the mechanism of action involved in the host-pathogen interaction. Considering the bacterium, *X. citri* strains are characterized by specific pathotypes (PthA4, PthA*, PthAw, PthB, and PthC), which are distinguished based on the conservation of repeated variable diresidues (RVDs), encoding transcription activator-like (TAL) effectors. These recognize the corresponding effector binding element (EBE) in the promoter of susceptibility plant genes, such as *LOB* and Sugar Transport TFs (Hutin et al., 2015). Genome editing of a single EBE allele (type 1) in the promoter of *CsLOB1* in ‘Duncan’ grapefruit (Jia et al., 2016) allowed the generation of transgenic lines resistant to a mutated Xcc strain, but susceptible to wild type-Xcc. Mutations in both EBEs alleles (type I and type II) of *CsLOB1* in “Duncan” grapefruit and “Wanjincheng” orange, result in reduced symptoms in transformed plants caused by wild-type Xcc infection (Jia et al., 2016; Jia et al., 2017b; Peng et al., 2017). Furthermore, to improve resistance through CRISPR/Cas9 approach on the *CsLOB1* promoter (Zhou et al., 2017), homozygous mutants have been generated directly from citrus explants decreasing the susceptibility to CBC knocking out the *CsWRKY22* a marker gene for pathogen-triggered immunity in ‘Wanjincheng’ orange (Wang et al., 2019). Moreover, the availability of Cas12a has been successfully used in *Citrus*. The efficiency of CRISPR/Cas12a has been examined for editing Cs*PDS* in ‘Duncan’ grapefruit *via* Xcc-facilitated agroinfiltration to modify two alleles of EBEPthA4-CsLOBPs. One of seven transformed ‘Duncan’ plants has been found to contain the highest mutation rate, demonstrating reduced canker susceptibility (Jia et al., 2019). Recently, Zhu et al. (2019) used *Fortunella hindsii*, a wild *Citrus* species characterized by a juvenile phase of about 8 months and a dwarf habit, to observe the effects of a successful CRISPR/Cas9 experiment using *Agrobacterium*-mediated transformation. Two gRNAs were synthesized to edit the *PDS* gene and five transgenic lines exhibited targeted mutagenesis sites, resulting in a global and mosaic albino phenotype (Zhu et al., 2019). These results suggest that editing or cisgenesis to induce early flowering could be a successful strategy to speed up gene characterization in functional genomic studies, especially for characters related to reproductive biology and to fruits.

Compared to genome editing approaches, there were fewer applications of cisgenesis in citrus. This was due to technical difficulties and limited knowledge on the function of specific genes and on their promoters. The only example in citrus involved the use of the Ruby gene, which is intronless, flanked by the CaMV 35S promoter and terminator (a classical intragenesis experiment), with the aim to produce ‘Mexican’ lime (*Citrus aurantifolia*) fruits highly enriched in anthocyanins content (Dutt et al., 2016).

It is noteworthy to remark that the application of NPBTs in citrus strictly relies on the availability of a transformation process that needs the use of selectable marker gene, in most of cases *nptII* gene. So far, no marker-free vectors have been used for cisgenesis and genome editing in citrus and this limits the possibility to apply NPBTs for these species.

### Transformation and Regeneration: The Main Bottlenecks of New Plant Breeding Techniques for Citrus Genetic Improvement

Regeneration and transformation represent the main bottlenecks for the use of molecular breeding and NPBTs, also in citrus. Stable or transient *Agrobacterium*-mediated transformation of juvenile tissues is the preferred approach to introduce foreign DNA, and has been performed on many citrus genotypes. To date, transformation and regeneration protocols are available for: ‘Carrizo’ citrange and ‘Swingle’ citrumelo (Cervera et al., 1998b), two rootstocks that can be considered model plants; ‘Pineapple’ (Peña et al., 1995), ‘Hamlin,’ and ‘Valencia’ sweet oranges (Dutt and Grosser, 2010); ‘Femminello Siracusano’ lemon (Gentile et al., 2007); ‘Duncan’ grapefruit (Jia et al., 2017b); clementine (Cervera et al., 2008), Mexican lime (Dutt et al., 2016); *F. hindsii* (Zhu et al., 2019). However, owing to the large number of important varieties belonging to different species and hybrids, the lack of specific protocols especially for commercial cultivars remains an issue.

Many factors affect the stable transformation process mediated by *A. tumefaciens* that usually involves explants pre-incubation, co-cultivation with the engineered bacteria, callus induction, and selection of the regenerated shoots. A balanced composition of plant growth regulators in the regeneration medium is important for inducing the proliferation of transformed cells. In particular, the presence of cytokinin in the callus-induction medium promotes callus formation (Guo et al., 2002; Khan et al., 2009). The regeneration rate is tightly correlated with photoperiod (Moreira-Dias et al., 2000; Marutani-Hert et al., 2012) and growth of non-transformed buds need to be avoided using the appropriate antibiotics (Ballester et al., 2008; Rodríguez et al., 2008). Furthermore, the rate of *A. tumefaciens* transformation is affected by explant source and type, and conditions by the duration of cocultivation (Dutt and Grosser, 2009). In ‘Tarocco’ orange tissues, a limited explant pre-incubation period with hormone-rich liquid medium was found to increase the transformation efficiency (Peng et al., 2019). Transient gene expression assays, performed *via Agrobacterium* infiltration in grapefruit leaves, have demonstrated that transient transformation efficiency can be influenced by different factors, such as infiltration medium, *Agrobacterium* concentration, and leaf development stage (Figueiredo et al., 2011). Li et al. (2017) showed that sweet orange leaves approaching full maturity were at an optimal, in terms of transient protein expression using Xcc-facilitated agroinfiltration (Jia and Wang, 2014).

**Figure 3:**
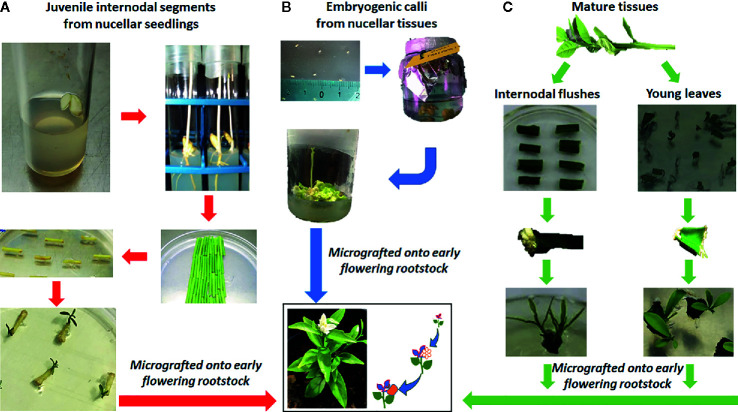
Different approaches for *Agrobacterium*-mediated transformation of citrus varieties starting from different tissue sources. **(A)** Juvenile internodal segments from nucellar seedlings. **(B)** Embryogenic callus obtained from unfertilized ovules. **(C)** Mature tissues, such as internodal segments from flushes and leaves. All the shoots or embryos, obtained from regeneration of transformed explants, are grafted onto vigorous rootstocks in order to induce early flowering, with the final aim to shorten the evaluation of the modified traits.

The choice between *in-vitro* juvenile and mature plants should also be considered, and it has to be approached differently if dealing with seedy or seedless fruits (**Figure 3**). Moreover, the polyembryonic nature of many citrus varieties enables the transformation of nucellar seedlings, leading to the production of transgenic plants that maintain the genetic background of the mother plant. Young tissues such as epicotyl explants (Peña et al., 1995; Dutt and Grosser, 2009) and leaf segments (Khan et al., 2009; Khan et al., 2012) have been widely used in the transformation mediated by *A. tumefaciens*, and high percentage of transformation efficiencies were achieved; however, plants regenerated from these tissues usually exhibit the long juvenility and require many years before bearing fruits, delaying the evaluation of horticultural characteristics of the transformed plants.

The use of explants from adult mature plants could overcome these limits through the use of *ex vitro* invigoration of source plant material, that consists in grafting mature buds onto juvenile rootstocks (Cervera et al., 1998a; Cervera et al., 2008; Orbović et al., 2015); however, the transformation efficiency of adult material, in citrus as well as in other fruit tree species, is several-fold lower than that obtained from juvenile citrus tissues (Almeida et al., 2003; He et al., 2011) genotype-dependent (**Table 1**) and experiment requires a high number of explants, together with high contamination rate that can complicate the process. Despite different efforts being made, the transformation efficiency of mature internode explants remains very low and does not exceed 6.1% (**Table 1**), except for ‘Hamlin’ sweet orange that shows 12.8% of transformed shoots (Orbović et al., 2015); the transformation of nodes with removed buds led to a transformation efficiency of 11.7% (Peng et al., 2019) and when thin section are used as explants, reaches 35% in ‘Pera’ sweet orange (Kobayashi et al., 2017). In addition, the different response of each cultivar to different regeneration conditions (Rodríguez et al., 2008) highlights the need to increase the number of citrus regeneration protocols; also in order to successfully apply transgenesis and NPBTs to citrus there is a need to optimize organogenic response of commercial varieties.

**Table 1:** List of genotypes, number of explants (internodes) from mature tissues, and transformation efficiency (TE%).

Genotype (species)	N° internodes cultured	TE%	Reference
Pineapple (*Citrus sinensis)*	294	6.1	Cervera et al., 1998a
Clementine (*C. clementina)*	600	3	Cervera et al., 2008
US-942	449	3.96	Marutani-Hert et al., 2012
(*C. reticulata* Sunki x *Poncirus trifoliata* Flying Dragon)			
Valencia (*C. sinensis)*	1,368	3.7	Fávero et al., 2012
Pera (*C. sinensis)*	1,104	1.4	Fávero et al., 2012
Hamlin (*C. sinensis)*	942	2.5	Fávero et al., 2012
Etrog (*C. medica)*	431	1.49	Marutani-Hert et al., 2012
Ruby Red (*C. paradisi)*	484	1.05	Marutani-Hert et al., 2012
Hamlin (*C. sinensis*)	530	12.8	Orbović et al., 2015
Pera (*C. sinensis*)	Not reported	35	Kobayashi et al., 2017
Tarocco (*C. sinensis)*	Not reported	11.7	Peng et al., 2019

An alternative to *Agrobacterium*-mediated transformation is the direct transformation of cells from suspension cultures obtained from citrus embryogenic calli derived from unfertilized ovules (Li et al., 2002; Dutt and Grosser, 2010; Omar et al., 2016) and of protoplasts (Guo et al., 2005; Dutt et al., 2018a; Omar et al., 2018).

These alternative strategies can be useful to transform recalcitrant genotypes and difficult-to-transform varieties such as some mandarin and lemon cultivars (Dutt and Grosser, 2010).

For both protoplasts and cells, the regenerated somatic embryos derive from a single cell, so that chimeras are rarely observed; on the other hand, this material requires longer time for the recovery of the regenerated plants and these will display juvenile nature (Dutt et al., 2018a). In citrus, the use of embryogenic cell culture and of protoplast technology has been exploited since 1990; many somatic hybrids have been generated through protoplast fusion (Grosser and Gmitter, 1990; Grosser and Gmitter, 2011; Grosser et al., 1992; Grosser et al., 2000) and transgenic plants have been recovered from transformed protoplasts (Guo et al., 2005; Omar et al., 2007; Dutt et al., 2018b; Omar et al., 2018) and calli (Dutt et al., 2018b). As for the application of NPBTs, citrus protoplasts could be used for a direct delivery of purified CRISPR/Cas9 ribonucleoproteins (RNPs) for efficient targeted mutagenesis, as already performed in grapevine and apple for transient cell transformation (Malnoy et al., 2016).

### Inducing Early Flowering as a Strategy to Shorten the Long Juvenility Phase in Citrus

The use of NPBTs in citriculture is hampered by limits in both the transformation and regeneration phases, as well as the long juvenility phase, which characterize citrus as well other fruit tree species (Hanke et al., 2007). Juvenility is adopted to reach faster a critical size to be able to compete in the environment. The photosynthesis products are directed toward the growth of the crown until the plant is not able to support the reproductive efforts (Hackett, 2011). Several approaches have been performed to active the precocious flowering in *Citrus*. The availability of a large germplasm collection and cross populations represent a source of precious material, which can be useful for individual natural early flowering plants. *F. hindsii* is a species that is phylogenetically close to the *Citrus* genus, which is particularly interesting because it produces flowers much earlier (around 8 months) than other common *Citrus* species (Zhu et al., 2019). In particular, *F. hindsii* needs around 5 months from seeds to T0 plantlets and about another 10 months to obtain the following T1 generation after the editing in *PDS* gene, supporting the evidence to be really promising using *F. hindsii* for functional studies (Zhu et al., 2019).

In the framework of biotechnological uses, it may be possible to reduce the duration of the breeding cycle acting on the endogenous genetic flowering pathways by using inducible promoters that activate transgene expression, in addition to several approaches used to transmit the transgenic stimulus through grafting (van Nocker and Gardiner, 2014). Evidence indicates that the genetic mechanisms of flowering are widely conserved among flowering plants (Albani and Coupland, 2010; Amasino, 2010), although the most promising is the FT/FTL1 family, encoding proteins that act as promoter or repressors of flowering. These proteins are highly similar to phosphatidyl ethanolamine-binding ones. These have been used widely in many woody plants, such as apple (Kotoda et al., 2010), plum (Srinivasan et al., 2012), and olive (Cerezo et al., 2014). In citrus for the first time Peña et al. (2001) used flowering involved genes (APETALA and LEAFY) to induce early flowering and fruiting. Later, Pons et al. (2014) highlighted the usefulness of the overexpression of early flowering genes as a tool to accelerate the evaluation of traits related to fruit quality. Moreover, the effect of grafting on the precious floral induction has been reported, up to now, in apple, where the M9 rootstock (a spontaneous mutant overexpressing *FT* genes) induces early flowering in juvenile apple scion (Foster et al., 2014). Similar results have been reported in *Arabidopsis*, tomato, and cassava (Lifschitz et al., 2006; Corbesier et al., 2007; Notaguchi et al., 2008; Silva Souza et al., 2018). In blueberry, the phenomenon was artificially reproduced using an FT transgenic rootstock and a non-transgenic scion (Song et al., 2019b).

Very recently, several examples have shown how the use of genome editing can also address the induction of early flowering, such as in kiwifruit (Varkonyi-Gasic et al., 2019). In tobacco the same approach was used to induce early flowering and precocious anthocyanin pigmentation (Liu et al., 2019). No published data are still available in citrus.

In plants, viral vectors have been used for the suppression of endogenous genes by virus-induced gene silencing (VIGS) and the expression of foreign genes (Senthil-Kumar and Mysore, 2011). An example is a viral vector based on the citrus leaf blotch virus (CLBV) developed by Velázquez et al. (2016). This vector carries the *FT* gene from *Arabidopsis thaliana* and *Citrus*. CLBV is a valid tool, because it does not produce symptoms in most commercial cultivars (Galipienso et al., 2000), it infects only the phloem (Agüero et al., 2013), and it is not transmitted by pests. Moreover, inoculated plants show normal reproductive biology producing flowers, viable pollen, and fruits (Velázquez et al., 2016). The viral vector cannot be integrated into the genome of the inoculated plant so is not transmitted by pollen (Vives et al., 2008). The CLBV vector carrying the *FT* gene anticipates flowering in young citrus plants at 4 months post-infection and for a period of at least 5 years, depending on the genotype and season. This vector is a biotechnological instrument with tremendous practical applications to speed-up traditional breeding programs and genetic studies.

Shortening the juvenile phase is useful for accelerating the selection process in citrus plants, in which resistance to pathogens is induced through the use of NPBTs. Recently, new sources of resistance to HLB have been identified in *Eremocitrus* and *Microcitrus* spp. (Ramadugu et al., 2016). The international citrus community is focused on the identification of specific genes that can be transferred using a cisgenic approach, and on the identification of susceptible genes to be edited. There is a risk of losing the world’s citrus crop if a solution is not promptly identified.

Use of the viral vector in combination with the NPBTs could substantially reduce the time needed to evaluate citrus plants edited for qualitative traits. Very few papers have reported the use of NPBTs to improve fruits or vegetables from a qualitative point of view. The major limitation of this approach is that the antioxidant, nutritional and healthy properties are under the control of several genes, generally multilocus, working cooperatively, producing complex, often under epigenetic control, not easy to be managed. From a genetic point of view, MYBs TFs have been widely reported to control polyphenol and carotenoid biosynthesis, in addition to other quality traits, such as flavor and texture, as reviewed in Allan and Espley (2018). In tomato, the concomitant overexpression of *Rosea1* (MYB) together with *Delila* (bHLH) produced very high levels of anthocyanin and purple fruit (Butelli et al., 2008). These engineered tomatoes were used in a mouse feeding trial and demonstrated a protective effect against cancer progression in Trp53–/– knockout mice (Butelli et al., 2008). Similarly, in apple, *MYB10* overexpression induced the large accumulation of anthocyanin in the flesh, and resulted in reduced inflammatory markers in mice (Espley et al., 2014). In *Citrus*, the slimming effect of pigmented Moro juice in obese mice (Titta et al., 2010) supported similar observations for other fruit, in addition to the fact that the *Ruby* gene is the MYB TF, which controls anthocyanins pigmentation in citrus fruits (Butelli et al., 2012; Butelli et al., 2017). The use of MYB TFs to enhance the levels of phytochemical levels in fruit, making them healthier, offers the potential to produce new crops that are not genetically modified. In this case, there is a need to observe the final phenotype in term of the quality and quantity of sugar, micro and macronutrients, vitamins, and seeds.

## Concluding Remarks

NPBTs are powerful tools used to improve quality traits without modifying the characteristics of elites in citrus, and to protect the world citriculture from the most aggressive and devastating disease, like HLB. *Citrus* species and relatives do not represent model species for the application of NPBTs due to the complexity of their biology; this is supported by a lack of confirmed data. To date, scientific research has placed scientists in a position to reduce the historical gap compared to other woody plants. Despite this, technical advances made in other woody species have involved the induction of plant regeneration from protoplast cells using the gRNA-Cas9/Cpf1 RNP complex in grapevine (Malnoy et al., 2016) as well as the release of CRISPR/Cas9 RNPs to induce DNA-free targeted mutations in apple and grapevine protoplasts (Osakabe et al., 2018); these data would be also be applied to citrus in order to establish more rapid transformation approaches. In particular, genome editing approaches could display their effectiveness especially for those characters for which the down regulation or the reduction of the expression levels of a single gene could determine a modification of a given trait.

Thus, overcoming genotype specificity and source explant problems, the availability of transformation platforms, and the adoption of new vectors and editing strategies, may provide citrus breeders reliable tools to quickly introgress genes related to fruit quality and other important agronomic traits.

## Author Contributions

FS prepared the main parts of the manuscript being the application of new plant breeding techniques in citrus the scope of his PhD project. AC focused on the paragraphs regarding methodologies for base editing, the contribution of horizontal gene transfer and on the induction of early flowering. LP and HP contributed to manuscript elaboration for the transformation and regeneration paragraph. SM and CL coordinated and wrote the manuscript.

## Funding

This work has been granted by Italian Ministry of Agriculture Food and Forestry through the project «BIOTECH Biotecnologie sostenibili per l’agricoltura italiana» (DM 15930/7305/2018).

## Conflict of Interest

The authors declare that the research was conducted in the absence of any commercial or financial relationships that could be construed as a potential conflict of interest.
